# Changes in cognitive domains during three years in patients with Alzheimer's disease treated with donepezil

**DOI:** 10.1186/1471-2377-9-7

**Published:** 2009-02-10

**Authors:** Cecilia M Persson, Åsa K Wallin, Sten Levander, Lennart Minthon

**Affiliations:** 1 Clinical Memory Research Unit, Department of Clinical Sciences in Malmö, Lund University, Lund, Sweden; 2 Department of Health and Society, Malmö University, Malmö, Sweden

## Abstract

**Background:**

The objective was to identify separate cognitive domains in the standard assessment tools (MMSE, ADAS-Cog) and analyze the process of decline within domains during three years in Alzheimer's disease (AD) patients with donepezil treatment.

**Method:**

AD patients (n = 421) were recruited from a clinical multi-centre study program in Sweden. Patients were assessed every six months during three years. All patients received donepezil starting directly after study entry. After dropouts, 158 patients remained for analyses over three years. Data for the other patients were analysed until they dropped out (4 groups based on length in study).

**Results:**

Factor analyses of all items suggested that there were three intercorrelated factors: a General, a Memory and a Spatial factor for which we constructed corresponding domains. Overall there was a cognitive improvement at six months followed by a linear drop over time for the three domains. Some group and domain differences were identified. Patients who remained longer in the study had better initial performance and a slower deterioration rate. The early dropouts showed no improvement at six months and many dropped out due to side effects. The other groups displayed a performance improvement at six months that was less pronounced in the Memory domain. Before dropping out, deterioration accelerated, particularly in the Spatial domain.

**Conclusion:**

The course of illness in the three domains was heterogeneous among the patients. We were not able to identify any clinically relevant correlates of this heterogeneity. As an aid we constructed three algorithms corresponding to the cognitive domains, which can be used to characterize patients initially, identify rapid decliners and follow the course of the disease.

## Background

Alzheimer's disease (AD) is a neurodegenerative disorder characterised by disturbances in many areas, including higher cognitive functions like memory, orientation and attention. AD is the predominant cause of dementia, a major source of disability and suffering among the elderly, and causing enormous and increasing economic costs for society [1-3]. At present, there is no effective cure available for AD. Symptomatic therapy, usually by means of choline esterase inhibitors (ChEI) is used on a regular basis. Short-term placebo controlled studies have reported small to moderate effect sizes of ChEI treatment on cognition in AD patients [4-7].

Almost all clinical trials on symptomatic therapy for AD use the Mini-Mental State Examination (MMSE) and/or the Alzheimer's Disease Assessment Scale – Cognitive subscale (ADAS-Cog). Most studies follow patients only over six months. Existing longitudinal studies support that the cognitive advantage gained by treatment established in short-term studies remains up to four years [8-14], however replications are required. Studies of more than three years often suffer from high dropout rates ranging from 67 to 96 percent [9-11]. What distinguishes patients that actually complete a three-year study from those who drop out, for example in pattern of cognitive reduction, needs to be further investigated.

AD is a disease that progresses through different brain layers, which implies changes in the cognitive symptom profile over time. One problem is that most studies on ChEI treatment do not present cognitive outcome measures divided into different domains [15]. To properly evaluate these treatments, it is important to assess changes in specific cognitive domains, mapped over time. Such data may provide hints as to the causal mechanisms and will have consequences for planning interventions for the individual patient, and for developing support systems for AD patients on the societal level. One standard way of grouping items into homogenous categories is by factor analysis. We have found only few factor analytic studies on MMSE or ADAS-Cog, reporting two or three factors typically reflecting general functioning, concentration, memory, language or praxis [16-20]. Comparisons among these factor analytic studies are difficult for technical reasons; mainly differences in the rotation procedures, and no authors have based their factor analyses on combined MMSE and ADAS-Cog data.

The present study aims at identify salient cognitive domains in the MMSE and ADAS-Cog instruments combined, and to describe patterns of change in patients with AD during three years of therapy by the ChEI donepezil. Furthermore we wanted to identify clinically relevant differences between 3-year completers and patients who dropped out before study end, including analysis of *APOE *genotype. We also wanted to construct algorithms within different cognitive domains, based on the patient material at study entry as a norm group. Such algorithms can be expected to be of use in the clinic as well as in treatment research.

## Method

The present study is part of the Swedish Alzheimer Treatment Study (SATS), an ongoing, prospective multi-centre study, designed to evaluate the long-term effect of ChEI treatment in patients with AD in a routine clinical setting. The SATS is run in ten memory clinics in Sweden, administrated from the Memory Clinic at U-MAS, Malmö. Presently more than 1,000 patients have been included in the study. Detailed information about the SATS study program is presented elsewhere [21].

### Subjects

This study is based on 421 AD patients recruited from the first 435 consecutive subjects with donepezil treatment included in the SATS study program between 1997 and 2001 (14 subjects were excluded due to missing values, v.i.). Inclusion criteria were clinical AD diagnosis according to the Diagnostic and Statistical Manual of Mental Disorders, 4th edition (DSM-IV) [22], and probable or possible AD, according to the criteria of the National Institute of Neurological and Communicative Disorders and Stroke and the Alzheimer's Disease and Related Disorders Association (NINCDS-ADRDA) [23], age above 40, living at home, either with a family member or having a caregiver. Exclusion criteria were other causes of dementia than AD, being already on ChEI treatment at study entry and contraindications to donepezil treatment. Other medications than antidementia ones were allowed, and the doses were recorded.

All participants and their caregivers provided written informed consent. The study was designed in accordance with the Helsinki Declaration and approved by the Ethics Committee at Lund University.

### Measures

#### MMSE

The MMSE was constructed to measure the cognitive aspects of mental status [24]. It includes ten items: Orientation, Registration, Attention (-Calculation), Recall, Naming, Repetition, Comprehension, Reading Ability, Writing Ability and Visual Construction. Sometimes the six latter are referred to as the Language category. The MMSE scale ranges from 0 to 30, the higher the score the better cognitive performance.

#### ADAS-Cog

The ADAS-Cog was developed by Rosen and co-workers [25] in order to measure cognitive aspects of AD. The standard ADAS-Cog scale includes eleven items, of which seven are short cognitive tests: Word Recall, Naming (objects and fingers), (Following) Commands, Constructional Praxis, Ideational Praxis, Orientation and Word Recognition, and four are scales rated by the clinician: Remembering Test Instructions, Spoken Language Ability, Word-finding Difficulty and Comprehension (of Spoken Language) [26]. The majority of studies with ADAS-Cog use the standard scale described above, ranging from 0 to 70. The version of ADAS-Cog used in the SATS study also comprises the items Delayed Word Recall and the rating scale Concentration (Distractibility) (max 85 points). In this study, all thirteen ADAS-Cog items were analysed as single items. If nothing else is stated, the standard eleven-item ADAS-Cog scale was used as a sumscore to enable comparisons to other studies. The higher the ADAS-Cog score the more cognitive impairment.

#### Genetics

The Apolipoprotein E genotype (*APOE*) has alleles with different combinations of variants ε2, ε3 or ε4. A pattern comprising one, or predominantly two, ε4 alleles has been suggested to be associated with AD. In this study, the variable *APOE *was dichotomous, as presence of at least one ε4 allele or not.

#### Duration of illness

Duration of illness was estimated by the treating physician through a clinical interview with the patient and caregiver about debut of the first AD symptoms, at their first visit at the memory clinic.

### Procedure

Before inclusion in the study, patients were thoroughly examined to rule out other causes of dementia than AD. Prior to donepezil (Aricept^®^) treatment, the patients were tested with a study entry battery, including MMSE and ADAS-Cog. Two months after study entry the patients were tested by MMSE a second time. Every six months after study entry, they were repeatedly tested by both MMSE and ADAS-Cog up to end of study (three years). Thus, patients were tested, at most seven times by ADAS-Cog and eight times by MMSE.

All decisions regarding medication, dosage etc. were made by the treating physician according to clinical judgment. At study entry, all subjects received 5 mg donepezil per day. Over time an increasing number of patients were treated by the maximum dose of 10 mg. After 12 months a majority received 10 mg.

### Statistical analysis

In a first phase of the analysis, data were scanned for missing values and outliers. Since our statistical analyses require complete results for all test sessions, missing values were replaced for patients having at most 30 percent missing values at MMSE and ADAS-Cog (up to their last test session before eventual dropout). Fourteen patients with more than 30 percent missing values were excluded, leaving 421 patients for analysis. For MMSE and ADAS-Cog sumscores, missing values were replaced only for 3-year completers (N = 158). All missing data were replaced by predicted values as the results of linear regression analyses based on the other test (MMSE/ADAS-Cog) at the same test session and earlier and later test results for both tests as independent factors (4.4% of all values in MMSE and 6.4% in ADAS-Cog were replaced this way; r varied between .80 and .97). Item scores were not replaced. Replacement of data for cognitive domains was performed for 3-year completers (with max 4/14 tests missing), for 2-year completers (max 3/10 tests missing) etc. Again, values were predicted by linear regression analyses based on the other tests items at the same session, earlier and later domain scores etc. (r varied between 0.95 and 1.0). In total 9.8 percent of the domain variables were replaced this way. Four patients were on one test session each judged as extreme outliers (the results differed a lot from earlier and later results). These values were replaced in the same way as described above.

Data were analysed by standard statistical methods (SPSS 15.0) and by latent class modeling analysis (Latent Gold 4.0, [27]). The analyses on MMSE and ADAS-Cog sumscores were based on the 3-year completers (N = 158), and results on item level on 118 subjects for MMSE and 100 subjects for ADAS-Cog, for which there were complete item data. Homogeneity was assessed by Cronbach's alpha, which is equivalent to an icc value as computed by SPSS 15.0. In order to define cognitive domains, principal component factor analyses were performed on all items (MMSE and ADAS-Cog combined), with oblique rotation (Promax), and checked by the latent class modeling factor analysis, which is non-parametric. Details are provided in the Results section. An alpha level of p < .05 was used for all statistical tests.

## Results

### Group characteristics and dropout analysis

**Figure 1:**
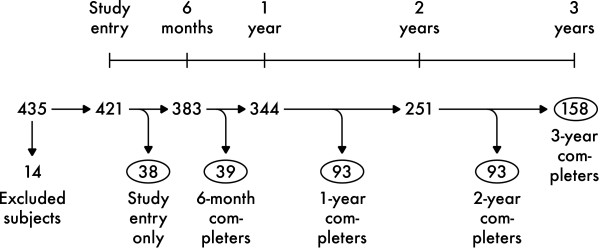
**Patient flow through the study, frequencies**. Circled numbers represent the groups based on length in study.

At study entry, 421 subjects were included. Over the following years patients dropped out as depicted in Figure 1, and for various reasons (v.i.). At study end 158 (38%) patients were available for analyses of changes in parameters over the whole time period.

Dropout causes were: admission to nursing home (24%), change of therapy (17%), suspected side-effect (13%), poor compliance (9.9%), deceased (9.5%), withdrawn informed consent (9.5%), intercurrent somatic disease (6.1%) and other causes (11%). For details regarding drop-out in the SATS, see Wallin et al. [21]. Dropping out due to side effects was strongly overrepresented among patients who participated only in the study entry test-session (χ^2 ^(4) = 84.8, p < .001); otherwise there were no significant effects in terms of reason for dropout.

Study entry characteristics of groups based on length in study (see Figure 1) are summarized in Table 1. The 3-year completers differed significantly from the whole group in terms of age, MMSE and ADAS-Cog, but not sex distribution, illness duration or *APOE *(t- and χ^2 ^tests). Numerically the differences in the means were small and clinically non-significant as defined by Cohen [28], except for ADAS-Cog (around 0.3 SD).

**Table 1:** Study entry characteristics for groups classified by length in study

Characteristic	Study entry only	6-month completers	1-year completers	2-year completers	3-year completers	Total SATS sample
N	38	39	93	93	158	435
Sex (men)	31.6%	25.6%	32.3%	45.2%	35.4%	35.2%
Age (years)	75.8 ± 4.67	77.7 ± 4.17	74.7 ± 6.21	74.2 ± 6.87	73.8 ± 6.99	74.6 ± 6.53
Duration (yrs)	3.43 ± 2.38	2.92 ± 1.53	3.08 ± 2.04	3.13 ± 2.15	3.11 ± 2.61	3.11 ± 2.26
*APOE *ε4 allele^a^	69.7%	60.0%	74.7%	61.5%	63.9%	66.0%
MMSE	21.7 ± 4.01	20.9 ± 4.49	21.6 ± 5.18	22.0 ± 4.21	23.1 ± 4.15	22.0 ± 4.55
ADAS-Cog (70)	22.9 ± 10.7	22.7 ± 9.26	21.9 ± 11.2	21.9 ± 9.89	18.2 ± 9.03	20.7 ± 10.0
ADAS-Cog (85)	32.6 ± 12.6	32.2 ± 10.9	30.7 ± 12.8	31.3 ± 11.4	26.8 ± 10.8	30.0 ± 11.8

Note. Values are presented as Mean ± SD or percent, ^a ^Percentage carrying at least one *APOE *ε4 allele.

These initial subject characterizations and the analyses in the following subsection on MMSE and ADAS-Cog are intended as a preamble to the main issue of this report, the identification and analyses of cognitive domains.

### MMSE and ADAS-Cog

Means ± SD for the MMSE sumscore, and medians and quartiles for the ten MMSE items, over time are presented in Table 2. The decline over the three years was 3.9 MMSE points, corresponding to an annual decline of 1.3 points. Homogeneity for the instruments is a measure of the "overall correlation among the items", it was analysed in order to study the statistical properties prior to the factor analyses (which are affected by the homogeneity). Homogeneity for the MMSE items was estimated by Cronbach's alpha for session 1, 5 and 8. For session 1 and 5, three items with extreme distribution were not entered. For session 1, alpha was .58, for session 5, it was .68 and for session 8 (all items included) alpha was .76. For all analyses there were no items that displayed a substantially lower correlation with the sumscore than the others.

**Table 2:** Central and variation measures for MMSE items and sumscores for the eight assessments

Test	Max score	Study entry	2 months	6 months	12 months	18 months	24 months	30 months	36 months
MMSE Items									
Orientation	10	8 (6, 9)	8 (7,10)	8 (6, 9)	7 (6, 9)	7 (5, 9)	6 (4, 8)	6 (4, 8)	6 (3, 8)
Registration	3	3 (3, 3)	3 (3, 3)	3 (3, 3)	3 (3, 3)	3 (3, 3)	3 (3, 3)	3 (3, 3)	3 (3, 3)
Attention	5	5 (3, 5)	5 (3, 5)	5 (4, 5)	5 (3, 5)	5 (3, 5)	4 (2, 5)	4 (2, 5)	3 (1, 5)
Recall	3	0 (0, 1)	0 (0, 2)	0 (0, 1)	0 (0, 1)	0 (0, 1)	0 (0, 1)	0 (0, 1)	0 (0, 1)
Naming	2	2 (2, 2)	2 (2, 2)	2 (2, 2)	2 (2, 2)	2 (2, 2)	2 (2, 2)	2 (2, 2)	2 (2, 2)
Repetition	1	1 (0, 1)	1 (1, 1)	1 (1, 1)	1 (1, 1)	1 (1, 1)	1 (1, 1)	1 (1, 1)	1 (0, 1)
Comprehension	3	3 (2, 3)	3 (3, 3)	3 (3, 3)	3 (2, 3)	3 (2, 3)	3 (2, 3)	3 (2, 3)	3 (2, 3)
Reading Ability	1	1 (1, 1)	1 (1, 1)	1 (1, 1)	1 (1, 1)	1 (1, 1)	1 (1, 1)	1 (1, 1)	1 (1, 1)
Writing Ability	1	1 (1, 1)	1 (1, 1)	1 (1, 1)	1 (1, 1)	1 (1, 1)	1 (1, 1)	1 (1, 1)	1 (0, 1)
Visual Constr.	1	1 (0, 1)	1 (0, 1)	1 (0, 1)	1 (0, 1)	1 (0, 1)	1 (0, 1)	0 (0, 1)	0 (0, 1)
MMSE Sumscores									
MMSE, men	30	23.4 ± 4.24	24.3 ± 4.34	24.1 ± 4.49	23.0 ± 5.62	23.0 ± 6.07	22.3 ± 6.56	21.7 ± 7.27	20.4 ± 8.12
MMSE, women	30	22.9 ± 4.12	23.8 ± 3.89	23.8 ± 3.66	22.8 ± 4.21	21.6 ± 5.12	20.6 ± 5.74	19.6 ± 6.31	18.6 ± 6.81
MMSE, total	30	23.1 ± 4.15	24.0 ± 4.05	23.9 ± 3.97	22.9 ± 4.74	22.1 ± 5.50	21.2 ± 6.07	20.4 ± 6.72	19.2 ± 7.33

Note. Items are presented as Median (quartiles 25, 75), sumscores are presented as Mean ± SD. n = 118 for MMSE items and n = 158 (56 men and 102 women) for MMSE sumscores.

Means ± SD for the ADAS-Cog sumscore and medians and quartiles for the eleven standard items plus the two additional items, over time are presented in Table 3. Cronbach's alpha for the ADAS-Cog data (all 13 items) for session 1, 4 and 7 was .79, .89 and .94, respectively.

**Table 3:** Central and variation measures for ADAS-Cog items and sumscores for the seven assessments

Test	Max error	Study entry	6 months	12 months	18 months	24 months	30 months	36 months
ADAS-Cog Items								
Word Recall	10	6 (5, 7)	6 (5, 7)	6 (5, 7)	7 (5, 8)	6 (5, 8)	7 (5, 8)	7 (5, 8)
Naming	5	0 (0, 1)	0 (0, 1)	0 (0, 1)	0 (0, 1)	0 (0, 1)	0 (0, 1)	0 (0, 1)
Delayed Word Recall^a^	10	9 (7, 10)	9 (7, 10)	9 (7, 10)	10 (7, 10)	9 (7, 10)	10 (8, 10)	10 (8, 10)
Commands	5	0 (0, 1)	0 (0, 1)	0 (0, 1)	1 (0, 1)	1 (0, 2)	1 (0, 2)	1 (0, 2)
Constructional Praxis	5	1 (0, 1)	1 (0, 1)	1 (0, 1)	1 (0, 1)	1 (0, 2)	1 (0, 2)	1 (0, 3)
Ideational Praxis	5	0 (0, 1)	0 (0, 1)	0 (0, 1)	0 (0, 1)	0 (0, 1)	0 (0, 1)	0 (0, 2)
Orientation	8	1 (0, 3)	1 (0, 3)	2 (1, 4)	2 (0, 4)	3 (1, 4)	3 (1, 5)	4 (1, 5)
Word Recognition	12	5 (3, 8)	5 (3, 8)	5 (3, 9)	5 (3, 9)	6 (3, 9)	6 (3, 9)	8 (5, 11)
Remembering Test Instr.	5	1 (0, 1)	1 (0, 1)	1 (0, 2)	1 (0, 2)	1 (0, 3)	1 (0, 3)	1 (0, 3)
Spoken Language Ability	5	0 (0, 1)	0 (0,1)	0 (0, 1)	0 (0, 1)	0 (0, 1)	0 (0, 1)	0 (0, 1)
Word-finding Difficulty	5	1 (0, 1)	1 (0, 1)	0 (0, 1)	1 (0, 1)	1 (0, 1)	1 (0, 1)	1 (0, 2)
Comprehension	5	0 (0, 1)	0 (0, 1)	0 (0, 1)	0 (0, 2)	0 (0, 1)	1 (0, 2)	1 (0, 2)
Concentration^a^	5	0 (0, 1)	0 (0, 1)	0 (0, 1)	0 (0, 1)	0 (0, 2)	0 (0, 1)	0 (0, 1)
ADAS-Cog Sumscores								
ADAS-Cog, men	70	17.5 ± 10.0	16.6 ± 9.44	17.7 ± 11.6	19.3 ± 13.3	20.4 ± 14.3	21.5 ± 15.9	23.6 ± 18.0
ADAS-Cog, women	70	18.7 ± 8.45	19.1 ± 8.99	20.4 ± 10.2	22.5 ± 11.6	23.7 ± 11.6	26.3 ± 14.1	29.5 ± 15.7
ADAS-Cog, total	70	18.2 ± 9.03	18.2 ± 9.21	19.5 ± 10.7	21.4 ± 12.3	22.5 ± 12.7	24.6 ± 14.9	27.4 ± 16.8

Note. The scores at ADAS-Cog reflect number of errors. Items are presented as Medians (quartiles 25, 75), sumscores are presented as Mean ± SD. n = 100 for ADAS-Cog items and n = 158 (56 men and 102 women) for ADAS-Cog sumscores. ^a ^The items Delayed word recall and Concentration are not included in the standard ADAS-Cog (max 70 points).

### Factor analyses and construction of cognitive domains

Factor analyses were conducted in order to objectively study how many, and the nature of, different dimensions (factors) that are detectable among the item scores, and which items that belong to (load on) those factors, to enable construction of domains based on those items. Multiple analyses were conducted to obtain more confident factor structures and to compare different time sessions.

In a first step, all MMSE and ADAS-Cog items for the same individual and session were entered into a database that contained 1867 such data sets. A factor analysis using Promax rotation was performed on that database. This analysis yielded three factors with Eigenvalues over 1 (10.6, 1.92 and 1.16 for factors 1, 2 and 3, respectively). Factor 1 was loaded by many variables and seems to reflect general cognitive performance, Factor 2 seems to reflect declarative memory, and Factor 3 seems to reflect spatial and praxis aptitudes.

Then, we performed similar factor analyses on the MMSE and ADAS-Cog items from each session at a time. The study entry session contained 421 complete cases, the following sessions a decreasing number of patients, with 146 cases in the last session. The pattern of Eigenvalues and factor loadings from the first two sessions were different from the following five, which yielded similar patterns as the comprehensive factor analysis. This probably reflects that those patients who dropped out early were different from the others.

All items displayed more or less deviant distributions making it questionable to use a parametric type of factor analysis. Results were therefore checked by applying the non-parametric, latent class modeling factor analysis. Findings were compatible, suggesting that the original decomposition of the variance into the three factors was valid.

We used the overall pattern to define to what domain each item could be referred to, using a factor loading of 0.40 as the lower limit. We computed new domain variables by summing z-scores for study entry MMSE and ADAS-Cog items according to which domain they belonged to, with equal weight for all items (except a few with less convincing association with a specific factor, which we gave half the weight). In order to obtain three comparable domain variables, we performed z-transformations of the summed z-scores, based on all subjects at study entry. These domain variables displayed rather strong intercorrelations, suggesting that the shared variance was around fifty percent. In the following text, these cognitive domain variables are denoted as (1) General domain, (2) Memory domain and (3) Spatial domain. Table 4 shows the association of each item with the three domains (see Appendix and Additional Files 1, 2and 3 for algorithms to compute these domains).

**Table 4:** List of MMSE and ADAS-Cog items that constitute the three cognitive domains

General domain		Memory domain		Spatial domain	
m2	Registration	m1	Orientation	m3	Attention
m5a	Naming	m4	Recall	m5c	Comprehension (.5)
m5b	Repetition (.5)	a1	Word Recall Task	m5e	Writing Ability
m5c	Comprehension (.5)	a3	Delayed Word Recall	m5f	Visual Construction
m5d	Reading Ability	a7	Orientation	a4	Commands (.5)
a2	Naming	a8	Word Recognition	a5	Constructional Praxis
a4	Commands (.5)			a6	Ideational Praxis (.5)
a6	Ideational Praxis (.5)				
a10	Spoken Language Ability				
a11	Word-finding Difficulty				
a12	Comprehension				
a13	Concentration				

Note. The code before each item indicates the succession number in our versions of the tests, m = MMSE and a = ADAS-Cog. The ".5" after an item indicates that the item contribute to its domain with half the weight.

### Cognitive domains over time

**Figure 2:**
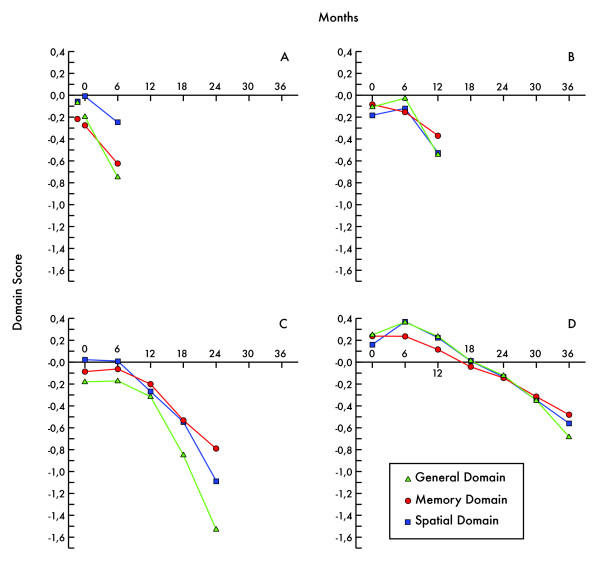
**Domain scores over time for AD groups based on time in study**. Scores are shown for patients only participating at study entry (N = 38) and 6-month completers (N = 39) (Subfigure A), for 12-month completers (N = 93) (B), for 24-month completers (N = 93) (C) and for 36-month completers (N = 158) (D). Domain scores represent z-scores based on the study entry session for all patients. In subfigure A, study entry patients are shown to the left with no lines.

The development over time measured by the three cognitive domains, for the subgroups defined according to time in study is shown in Figure 2. Inspection of the diagrams suggests the following conclusions. The shorter a subject remains in the study, the more poor appears the domain scores at study entry to be, particularly the General and the Memory domain scores. The Memory domain seems to differ from the others by displaying no clear inverted U-shape (quadratic effect) during the first year. The downward slope of the lines appears to be less steep for the 3-year completers. The other groups seem to accelerate in deterioration before dropout.

These visual "conclusions" are tested below, by analyses of variance (ANOVAs). Post hoc analyses are conducted to analyse which groups differ significantly from each other.

For analysis of the three domains at study entry, one-way ANOVAs were used to compare the groups based on length in the study. The groups differed significantly in the General and Memory domains (F(4, 420) = 4.02, p < .01 and F(4, 420) = 3.90, p < .01, respectively). Post hoc analyses (with S-N-K protection for mass significances) were non-significant, except for the Memory domain (3-year completers differed from those who did not complete even one year).

The development in the three domains over the first year was analysed by one-way ANOVAs for repeated measures (study entry, six months, one year) for the groups (1-year, 2-year and 3-year completers). For all domains, there were both a linear (i.e. worsening over the year) and a quadratic effect (i.e. a curve shaped like an inverted U). For the General and Spatial domains the quadratic effect was largest (F(1, 341) = 23.9, p < .001 and F(1, 341) = 38.3, p < .001, respectively). For the Memory domain, the quadratic effect was less pronounced (F(1, 341) = 8.56, p < .01). There was also an interaction between group and development over time for the General and Spatial domains (p < .01 and p < .001, respectively), but not for the Memory domain. Thus, the results for analyses over the first year suggest that the cognitive performance as measured by the General and Spatial domains was relatively good at six months (the quadratic effect); in these domains the groups also differed in development over time.

Similar analyses of the regression lines from Year 1 to Year 2 for the 2-year and 3-year completers, for the three domains, suggested that there was a highly significant and linear deterioration over time for both groups and all domains. However, for all domains, there were significant differences between the 2-year and 3-year completers in both level and slope.

Slope data, expressed as annual drop in domain scores over the last six months before eventual drop out, for groups according to time to drop out are provided in Table 5. Comparisons among the groups, domain by domain, by one-way ANOVAs showed that there were significant differences only for the spatial domain (F(3, 382) = 3.87, p < .01). A post-hoc analysis was marginally significant (p = .052), and became significant if the 6-month completers were omitted from the analysis (3-year completers differed from the other groups). The results verify the statistical robustness of the visual impression of the diagrams.

**Table 5:** Drop in the cognitive domains during the last six months for the groups

	Domain		
	
Group	General	Memory	Spatial
6-month completers	1.106	0.700	0.477
1-year completers	1.032	0.428	0.809
2-year completers	1.361	0.520	1.078
3-year completers	0.669	0.330	0.426

Note. Slope is expressed as annual drop in z-scores.

## Discussion

The patient group of this study can be characterized cognitively as non-advanced in their AD (study entry mean MMSE was 22). The homogeneity analyses of MMSE and ADAS-Cog suggested that (a) items are reasonably homogenous, limiting the possibility of identifying cognitive profiles of individual subjects based on such indices, and (b) the instruments, particularly MMSE, work better for groups that are more advanced in their cognitive reduction than the current group was at study entry. Still there was a substantial heterogeneity among subjects based on items. Compared to previous studies, MMSE was somewhat less and ADAS-Cog was somewhat more homogenous in our sample [26,29-32].

Even though homogeneity was high, a series of oblique factor analyses on all MMSE and ADAS-Cog items together yielded evidence for the presence of three reproducible factors, which also appeared to be clinically relevant. On the basis of the analyses, three cognitive domains were constructed, (1) General domain, (2) Memory domain and (3) Spatial domain.

The longer a subject remained in the study, the better were the General and Memory domain scores at study entry. The 6-month completers did not improve cognitively in any domain. In the other groups, there was a six month peak (operationalized as the quadratic component of the effect of time during the first year, v.i.) in all domains, but the effect in the Memory domain was less pronounced. The reduction in performance from 12 to 24 months differed markedly between the 2- and 3-year completers even if both groups deteriorated over time. A closer look at the final six months before dropout in the different groups of subjects suggests that once the speed of deterioration within the Spatial domain is large enough, around 1 domain score per year, dropout is imminent.

The study is a prospective, naturalistic one, with virtues and limitations typical of such studies. One virtue is the representativity of patients seen in clinical practice and thereby the potential for generalizability of the findings. On the other hand, generalizability is compromised by the substantial dropout rate; only 38% of the patients could be followed over the three years. However, this is not an unusually high dropout rate considering the illness studied [9-11].

The three-year completers were younger and had better cognitive scores at baseline. According to Wallin et al. [21], who reported on the same material, those who dropped out the first year were older, had lower MMSE at baseline but did not differ in gender, duration, *APOE *or ADAS-Cog at baseline.

The multiple causes of dropout makes it complicated to analyse the clinical implications of differences between groups according to length in study. Our intention was to present our data as an example of cognitive course of AD subjects in a clinical longitudinal study. There were substantial differences in the domain scores (level and rate of decline) between the groups. These differences were present already at study entry, but became larger over time and most accentuated in the six months period before dropout. This suggests that it is not necessarily so that a study group followed for a long period is representative for the whole sample even if it does not differ at study entry.

The construction of domain scores based on factor analyses of MMSE or ADAS-Cog is not common in the literature, neither are factor analyses in general. Many researchers have probably been discouraged to involve in such analyses because of the high homogeneity of these instruments as well as the skewed distributions on item level. Still, we performed parametric analyses and were able to identify three correlated but reproducible factors, in the combined MMSE and ADAS-Cog data set. The factor pattern is similar to patterns obtained in previous factor analytic studies of separate MMSE or ADAS-Cog data using oblique rotation [16,19,20]. Oblique rotation accepts correlated factors, which are required due to the high homogeneity among the items, probably resulting in factors more representative for the subject characteristics. We checked the representativity of the findings using a new non-parametric type of factor analysis based on latent variables [27]. We created domains by summing standardized item scores rather than using factor scores [20].

The segregation of the domain data over time suggests that the factor analyses and subsequent allocation of items to domains generated valid information. However, the lack of similar analyses in the literature suggests that the findings must be cross validated before they can be regarded as robust.

An alternative would be to construct cognitive domains based on a comprehensive neuropsychological battery, which might result in more informative and less correlated domains. However, as the cognitive deterioration becomes more advanced, such assessment batteries may become difficult to administer. Another reason to try to base domains on MMSE and ADAS-Cog is that they are so widespread and fast and easy to administrate; domains constructed from them have potential to be of good use in clinical practice as well as research.

In most groups there was a peak of the performance at six months, the six month assessment was above the line between the study entry and the twelve month assessments, see Figure 2. We assumed that an untreated group of AD patients would not suddenly improve their cognitive functions on group level; therefore we assumed that this six month peak may be regarded as an effect of treatment (including placebo effects and possibly some practice effects). Since "treatment effect" may not be an adequate expression lacking a control group, we just denoted the phenomenon "six month peak". This six month peak may be described as an inverted U-shape, statistically operationalized as the variance contribution of the quadratic effect over the first year estimated by an ANOVA for repeated measures.

One interesting finding is that the Memory domain did not peak at six months as much as the other domains, and also in other respects differed from the General and the Spatial domains. One possible explanation is that the disease process has gone further with respect to memory (hippocampal) functions already before initiation of treatment than for cognitive domains with predominantly neocortical localization. This theory has received some support by earlier research [20,33]. For instance, memory and spontaneous language items appear to be the earliest indicators of AD, while praxis, commands and naming items were sensitive later in the course of the disorder. It is also evident from Tables 2 and 3 that memory items are relatively impaired already at study entry. There may be other mechanisms responsible for degeneration in hippocampal structures, linked for instance to NMDA neurotoxicity, which are potentially amenable by drugs such as memantine [34]. This is a good reason to use memory assessment techniques that better reflects hippocampal dysfunction.

As the cognitive domains are standardized based on the study entry session data, an early relative impairment in memory items, results in a Memory domain with less potential for low scores. That is a negative consequence of using clinical norm data and a reason not to be uncritical when comparing the domains with each other, even though they are standardized and based on the same data set.

We have no good explanation why patients, who at study entry did not differ much, could follow such different courses. Turning the former argument up side down, the deteriorating speed could become a useful clinical index.

Researchers have proposed definitions of rapid cognitive decline in AD patients, based on the MMSE or ADAS-Cog sumscores. However, such measures do not always correspond to clinical decline as measured by other instruments [35,36]. One alternative would be to construct indices of cognitive decline within separate cognitive domains rather than on sumscores, and based on as much information as possible. We provide algorithms (Appendix) for the three domains, which enable a clinician to characterize an individual patient with respect to her/his current status, relative our patients at study entry, and compute slopes reflecting the speed of cognitive change. In this way, better criteria (based on more information and more homogenous information) can be established, for instance for "rapid decliners" [36] .

As stated above, only few researchers have applied factor analysis to MMSE and ADAS-Cog data. An alternative to the factor analytic approach would be to directly focus patient heterogeneity by using cluster analysis. In line with this we will report on a re-analysis of the present material using a non-parametric latent class cluster analysis (Persson, Wallin et al, forthcoming).

## Conclusion

The main finding of the present study is that MMSE and ADAS-Cog data reflect three intercorrelated cognitive domains, which differ in terms of the development over time and possibly among patient groups. The overall development of AD patients in this study included a cognitive improvement at six months after ChEI onset that was less pronounced in the Memory domain, followed by a linear drop over time for the three domains. Groups who remained longer in the study had somewhat better initial performance and increased this advantage substantially over time. This trajectory might reflect dropout mechanisms rather than being a characteristic of groups of AD patients with better prognosis. We provide algorithms and an SPSS syntax file (see Appendix and additional files 1 to 3) for calculation of the three cognitive domains, which may be used to assess the current status, decline rate or drug treatment outcome in patients at clinics or included in studies, against the background of the SATS material. The character of the SATS material is such that it can be regarded as a kind of norm material of AD patients in a rather early phase of the disease.

## Competing interests

SL has received fees from Lundbeck A/S for lecturing on antipsychotic drugs. Otherwise, the authors declare that they have no competing interests.

## Authors' contributions

CMP participated in the design of the study, analysed and interpreted data and drafted the manuscript. ÅKW contributed to the interpretation of data and critically revised the manuscript. SL participated in the design, analysed and interpreted data and helped to draft the manuscript. LM conceived of the study and participated in its design. All authors read and approved the final manuscript.

## Appendix: algorithms for calculation of cognitive domains

For SPSS users, a syntax file that can be used to automatically calculate domain scores (and replace ADAS-3 and ADAS-13) is available at the BMC Neurology website (see additional files 1, 2 and 3).

To compute domain scores manually please start by calculate z-values for the items with this algorithm:

z-item_x _= (item_x _- a_x_)/b_x_

where a and b for each item are found in Table 6.

**Table 6:** Item notations, variables for calculation of z-values and domain belongings

Item	Notation	a	b	Domain belonging
MMSE				
Orientation	mmse1	7.39	2.19	2
Registration	mmse2	2.90	0.413	1
Attention	mmse3	3.47	1.74	3
Recall	mmse4	0.559	0.897	2
Naming	mmse5a	1.97	0.178	1
Repetition	mmse5b	0.756	0.430	1(.5)
Comprehension	mmse5c	2.62	0.659	1(.5) and 3(.5)
Reading Ability	mmse5d	0.975	0.157	1
Writing Ability	mmse5e	0.841	0.366	3
Visual Construction	mmse5f	0.563	0.497	3
ADAS-Cog				
Word Recall Task	adas1	6.36	1.68	2
Naming	adas2	0.626	0.982	1
Delayed Word Recall	adas3	8.31	2.10	2
Commands	adas4	0.779	1.07	1(.5) and 3(.5)
Constructional Praxis	adas5	0.955	0.896	3
Ideational Praxis	adas6	0.636	0.979	1(.5) and 3(.5)
Orientation	adas7	2.18	1.99	2
Word Recognition	adas8	5.69	3.16	2
Remembering Test Instructions	adas9^a^			
Spoken Language Ability	adas10	0.541	0.794	1
Word-finding Difficulty	adas11	0.876	0.942	1
Comprehension	adas12	0.812	0.927	1
Concentration	adas13	0.729	1.00	1

Note. ^a ^adas9 is not included in any domain. "(.5)" after domain belonging indicates half weight.

Then, use the resulting z-items with the following algorithms to calculate domain scores:

General domain (1) = (zmmse2 + zmmse5a + 0.5·zmmse5b + 0.5·zmmse5c + zmmse5d - zadas2 - 0.5·zadas4 - 0.5·zadas6 - zadas10 - zadas11 - zadas12 - zadas13)/5.60

Memory domain (2) = (zmmse1 + zmmse4 - zadas1 - zadas3 - zadas7 - zadas8)/4.43

Spatial domain (3) = (zmmse3 + 0.5·zmmse5c + zmmse5e + zmmse5f - 0.5·zadas4 - zadas5 - 0.5·zadas6)/3.53

### Replacement of ADAS-3 and ADAS-13

Since the standard ADAS-Cog (max score 70) does not comprise the items ADAS-3 and ADAS-13, we have provided simple algorithms to compute values that can replace them.

Algorithm for replacement of ADAS-3:

adas3 = 1.2·adas1 - 1.3·mmse4 + 2.2

Round to integer between 0 and 10 (e.g. the result 11.8 should be rounded to 10)

Algorithm for replacement of ADAS-13:

adas13 = 0.38·adas11 + 0.77·adas12 - 0.42

Round to integer between 0 and 5

These replacement algorithms were constructed aided by step-wise linear regression analyses with all MMSE and ADAS-Cog items as possible predictors (independent variables) in a file with all complete test sessions for all patients (n = 1887). The correlation between predicted and actual value was r = .76 for ADAS-3, and r = .70 for ADAS-13.

## Pre-publication history

The pre-publication history for this paper can be accessed here:



## Supplementary Material

Additional file 1 **Cognitive Domains Syntax.** An SPSS syntax file to be used for automatic calculation of cognitive domains. Click here for file

Additional file 2 **Cognitive Domains Template.** An SPSS data file to be used together with the syntax file, a template in which MMSE and ADAS-Cog item scores are to be entered. Click here for file

Additional file 3 **Cognitive Domains Instruction.** A text file with instructions on how to use the syntax and template files. Click here for file
